# Development of a clinical protocol for detection of cervical cancer precursor lesions

**DOI:** 10.1590/1518-8345.2340.2999

**Published:** 2018-05-17

**Authors:** Deise Maria Do Nascimento Sousa, Ana Carolina Maria Araújo Chagas, Camila Teixeira Moreira Vasconcelos, Airton Tetelbom Stein, Mônica Oliveira Batista Oriá

**Affiliations:** 1 MSc. in Nursing. Doctor Degree Student. RN, Universidade Federal do Ceará, Fortaleza, CE, Brazil. Deise Maria do Nascimento Sousa- Bolsista CAPES - CNPq 479373/2013-2.; 2 MSc. in Nursing. Doctor Degree Student. RN, Universidade Federal do Ceará, Fortaleza, CE, Brazil.; 3 PhD. in Nursing. Adjunct Professor. RN, Universidade Federal do Ceará, Fortaleza, CE, Brazil.; 4 PhD. Medical Sciences. Professor. Collective Health, Universidade Federal de Ciencias da Saúde de Porto Alegre, Porto Alegre, CE, Brazil.; 5 PhD. in Nursing. Adjunct Professor. RN, Universidade Federal do Ceará, Fortaleza, CE, Brazil.

**Keywords:** Cervical Intraephitelial Neoplasia, Clinical Protocol, Validation Studies as Topic, Cervical Cancer Prevention, Women’s Health, Nursing

## Abstract

**Objective:**

to develop and validate the content of a clinical protocol aimed at prevention of cervical cancer in primary care.

**Method:**

technological research according to the steps: (1) submission of the project to the research ethics committee; (2) bibliographic survey; (3) elaboration of the clinical protocol; and (4) content validation. In the third step, the information was collected through bibliographic research and gynecology specialists were consulted. For the final step, four judges were selected to evaluate the clinical protocol according to AGREE 2. Domains that reached the minimum level of agreement of 75% in the scores were considered validated.

**Results:**

the scores obtained in each domain of the instrument were as follows: domain 1 (scope and purpose) = 87.5%; domain 2 (stakeholder involvement) = 83.3%; domain 3 (development rigor) = 79.7%; domain 4 (clarity of presentation) = 76.3%; domain 5 (applicability) = 78.1%; and domain 6 (editorial independence) = 85.4.

**Conclusion:**

the clinical protocol proved to be a validated material with scores above the minimum required. The protocol obtained positive recommendations with modifications and went through adjustments in order to make it more effective.

## Introduction

It is estimated that the number of cases of cervical cancer worldwide reaches 527,600, and this disease is responsible for 265,000 deaths^1^. In Brazil, data show that 15,590 new cases are diagnosed each year, corresponding to an estimated incidence of 15.33/100,000 inhabitants. Moreover, it is estimated that 5,160 of the confirmed cases of the disease result in death. Among the regions of the country, the North has the highest incidence of the disease, with 23.57 cases/100,000 women, followed by the Center-West and Northeast with 22.19/100,000 and 18.79/100,000 women, respectively; in the fourth position is the South, with a rate of 15.87/100,000 women, and in the fifth position, the Southeast with a record of 10.15/100,000 women. It is believed that 930 new cases occur in the state of Ceará, and 280 are expected to occur in the city of Fortaleza, with gross incidence rates of 20.27 and 20.53/100,000, respectively^2^.

In view of this epidemiological scenario, screening for precancerous lesions in the cervix is ​​a secondary prevention strategy in relation to cervical cytology and Pap smears. It is recommended that they be performed mainly in women aged 25 to 64, with a frequency of once every three years in the case of two consecutive Pap smears with negative results. To ensure the effectiveness of this test, it is necessary that there be a coverage rate of at least 80% of the population. This can directly interfere with mortality from cervical cancer, reducing the death rate by half^3^ ^-^ ^4^.

Considering that screening actions are the main source of evidence for detection of cervical cancer precursor lesions, it is necessary to build a protocol to be followed by nursing professionals during gynecological consultations. This protocol will provide greater support to their practice and to contribute to the early detection of precursor lesions and consequent decrease of the incidence of cervical cancer, as well as promote a better quality of care to clients.

Nurses play a fundamental role in consolidating the adequate coverage of cervical cancer prevention. They are among the professionals who are responsible for its realization and for encouraging the adherence of users to the follow-up and to appropriate periodicity of the examination. They also perform health promotion activities that aim to educate patients about the risk factors of the disease, as well as increase the number of adherents to regular visits to the Pap smear test^5^ ^-^ ^6^.

In this way, the creation of protocols to direct the care practices and routine procedures of professionals in diverse services becomes fundamental for its organization and management. It is worth mentioning that all the actions advocated in this type of material are prepared by specialists in the area of action to which it is proposed and these are based on the best scientific evidence. When it comes to application in the health area, they are known as clinical protocols or clinical guidelines, for they are directed to the search for quality and promotion of the user’s health, focused on preventive actions such as the Pap smear test^7^.

Because it is a type of technology, clinical guideline are recommended to be used in the screening of cervical cancer, providing greater appropriation of the health problem that is reported, allowing professionals to have technical and scientific support backing their actions, favoring greater self-confidence in their practices^7^.

In view of the above, the objective of this study was to develop and validate the content of a clinical guideline aimed at gynecological nursing consultation for prevention of cervical cancer in primary care.

## Method

This is a research of technological development in health^8^ carried out in four steps: (1) submission of the project to the research ethics committee; (2) bibliographic survey; (3) elaboration of the clinical protocol; and (4) content validation.

The step of preparation of the clinical guideline included the following phases: an integrative review ^9^ in the databases LILACS (Latin American and Caribbean Health Science Literature), PubMed (*Public/Publish Medline*) CINAHL (*Cumulative Index to Nursing and Allied Health Literature*), *Web of Science, Science of Direct* and *Cochrane*, using the following guiding question: *Which are the most accurate screening methods for early detection of cervical cancer lesions in women with active sex life?* As inclusion criteria, complete research articles, published in Portuguese, English or Spanish ​​and portraying interventions used to screen for cervical cancer were included in the survey.

**Figure 1:**
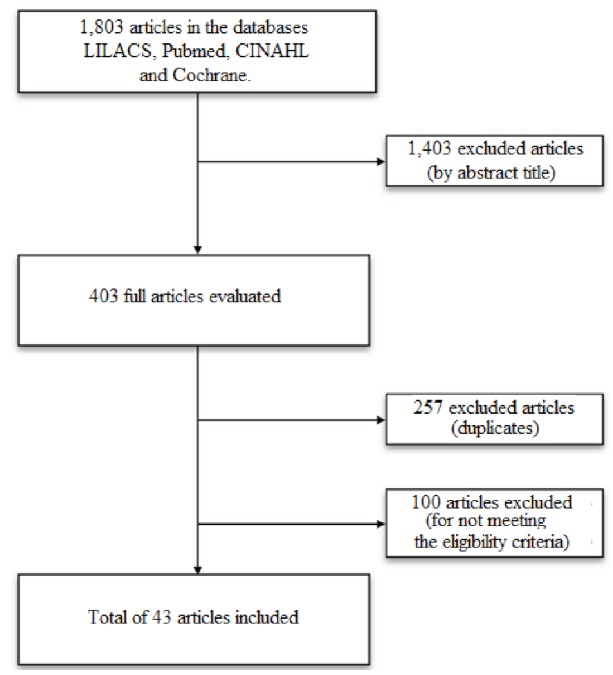
Mechanism of search in the integrative review. Fortaleza, CE, Brazil, 2014

Due to the specific characteristics of the access to each of the six selected databases, the strategies used to locate the articles were adapted to each database, having as a guiding axis the previously established question and the inclusion criteria to maintain consistency in the search of articles and avoid possible biases. The key words were the controlled descriptors: Cervical Cancer, Papillomavirus Infections and Pap smear Test. Keywords that are not controlled descriptors were also used, namely: colposcopy, cervicography, visual inspection with acetic acid, visual inspection with iodine and lugol. Six searches were performed at each base, using different combinations between the mentioned descriptors. The search was performed by online access, in February 2014, and the final sample of this integrative review was composed of 43 articles, according to Figure 1.

During the evaluation of articles, an instrument adapted from the literature was used to extract the data^10^, including the following items: identification of the original article; methodological characteristics of the study; evaluation of methodological rigor; interventions measured; and the results found in the study. For analysis and subsequent synthesis of the articles that met the inclusion criteria, a synoptic table was used to present data, also adapted and specially prepared for this purpose, including the following aspects: title of the research; authors’ name; intervention studied; results; recommendations; and conclusions^10^. It is noteworthy that during the elaboration of the clinical guideline, the levels of evidence and degrees of recommendation were used to classify the evidences found^11^.

Clinical decisions contained in the guideline were represented in the form of algorithms. This type of representation facilitates the understanding of professionals^7^. For the editing and organization of the algorithms, the *Microsoft Visio* *2013* software was used. The references used in the elaboration of the clinical guideline were arranged in *Vancouver* format. After completing all these steps, the guideline was sent to the duly specialized professional to review the Portuguese. The *guideline* developed in this study was recorded in the ISBN (*International Standard Book Number*).

An instrument of international use was applied for content evaluation. This instrument, called AGREE II (*Appraisal of Guidelines for Research and Evaluation*), aims to measure the methodological rigor and quality of clinical guidelines. In addition to conducting an overall assessment of the guideline, the AGREE II aims to provide a rigorous methodological strategy for the development of guidelines and to inform how the content of these guidelines should be presented in a clinical guideline. This tool recommends the participation of four (04) specialists to evaluate the quality of the guideline, selected by means of non-probabilistic sampling technique^12^. Invitations were sent to 04 gynecology specialists, from different professional categories, as recommended by AGREE II for a good evaluation of the clinical guideline. They were chosen according to pre-established criteria^13^.

After meeting the inclusion criteria, the specialists were invited to participate in the study through formal contact via invitation letter. At the same time, the evaluation questionnaire, instructions about the objectives of the study, and instructions for the adequate completion of the instrument were given to the specialists. After accepting to participate in the research, the informed consent term (ICF) was sent to professionals to register their consent.

**Figure 2:**
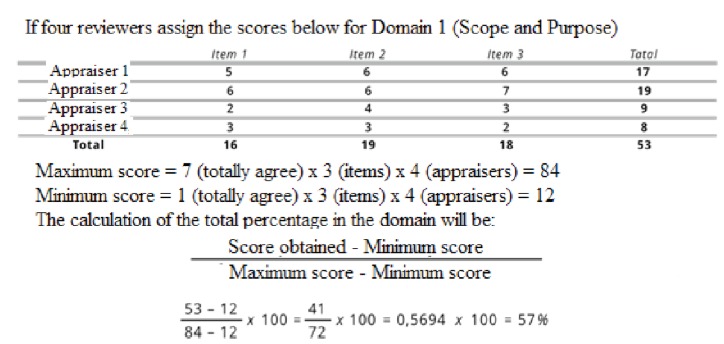
Example of Calculation of AGREE * II Score. Fortaleza-CE, April, 2017. Source: AGREE II Consortium. Instrument for evaluation of clinical guidelines: AGREE II (2009)

Data analysis was performed by calculating the adequacy of the clinical guideline proposed by AGREE II itself. Domain scoring is calculated by summing all scores of individual items in each domain and staggering the total as a percentage of the maximum possible score for each domain^14^ as shown in Figure 2.

*AGREE - Appraisal of Guidelines for Research & Evaluation

The overall assessment of the clinical guideline requires the specialist and/or appraiser to take into account the qualitative criteria considered in the evaluation process so that he can recommend its use; the assessment ranges from 1 to 7 on a *Likert*-type scale. The score given by each expert was tabulated in a *Microsoft Excel 2013* spreadsheet and the calculations were performed according to AGREE II, afterwards creating charts and tables. AGREE II does not determine the ideal cutoff point for the clinical guidance to be considered valid. However, the researchers adopted a 75% adequacy percentage in each evaluation performed to consider the protocol as validated.

This study was approved by the Research Ethics Committee of the Federal University of Ceará, under Opinion nº 401,240.

## Results

The evaluation of this clinical guideline was performed by four health professionals, who were named A1, A2, A3, A4. All of them work in the area of ​​gynecology and/or development and evaluation of health technologies; there were 02 physicians and 02 nurses who work in the area of ​​assistance and teaching. The time elapsed after graduation ranged from 7 to 30 years; two of them had a specialist degree, one had completed the Post-doctorate, and one had a Master’s degree.

**Figure 3:**
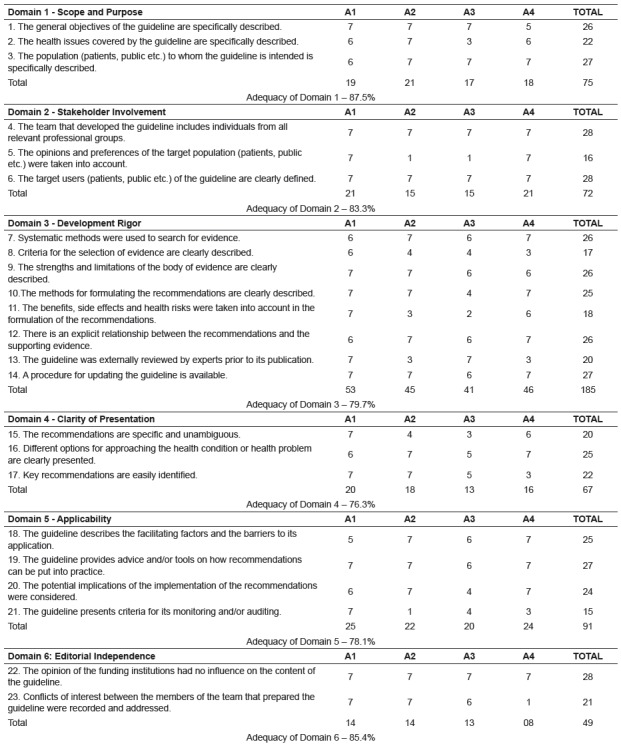
Distribution of the scores and suitability of the protocol according to the AGREE * II domains. Fortaleza (2017)

The assessment of adequacy of the clinical guideline was carried out through the AGREE II domains, presented in Figure 3.

* AGREE - Appraisal of Guidelines for Research & Evaluation

We found that the domain 1 (scope and purpose) obtained the highest score (87.5%). The domains 2 (stakeholder involvement) and 6 (editorial independence) also held scores above 80%. The domains 5 (applicability) and 3 (development rigor) achieved adequacy above 78%. The domain 4 (clarity of presentation) was the one that showed the lowest adequacy, 76.3%. It can be observed that all domains exceeded the minimum value of adequacy proposed by the authors.

The second item addresses whether the principal health issues are described in detail in the guideline through key questions. In this regard, A3 attributed 3 points indicating disagreement with the inclusion of digital cervicography in the clinical guideline, because this tool is little used in health services and has a low degree of recommendation. The other experts attributed 7 and 6 points and made no suggestion.

The third item analyzes whether the guideline has a clear description of the target population of the study, including variables such as sex, age group and clinical description. A3 suggested that the age of screening should receive more emphasis in the protocol, which was accepted; the guideline describes the age of onset of the screening, as well as the justification for it.

In relation to the *domain 2*, the appraisers A2 and A3 attributed only 1 point to item 5, justifying that the participation of the target public had not been included in the guideline. However, the authors clarified that the target public does not have sufficient expertise to make considerations during the gynecological consultation; during the anamnesis, the users were asked if they would be willing to perform other tests besides the routine test in order to identify more accurately possible cervical alterations, for which a positive answer was given.

Regarding the *domain 3*, specifically in relation to the item 8, A2 and A3 scored 4 points to this item, but they did not justify their score and did not make recommendations. A4 attributed 3 points, suggesting that the text should be brief and concise, and the paragraphs should be shortened. A3 attributed 4 points to the item 10, but did not provide any comment on this decision, contrary to the other experts who totally agreed on the clarity and presence of content in this topic of the guideline. In the item 11, A2 and A3 justified the low score by stating that the side effects and health risks were not clearly expressed throughout the text of the clinical guideline. The authors accepted this observation and revised the text of the clinical guideline in order to avoid any doubt about this aspect. A3 suggested revising the ASC-US (Atypical Squamous Cells of Undetermined Significance) algorithm, which should be further detailed. This suggestion was accepted by the authors. A4 questioned whether there would be any further evaluation after this validation process. However, it was clarified that, initially, there would be no further evaluation for this version of the clinical guideline. A new evaluation will be performed only when the tool is to be updated, what is scheduled to be done every three years with the possibility of anticipation whenever there is important clinical evidence to be added to this guideline.

In the *domain 4*, the specialist A2 suggested that the algorithm related to pregnant women should be excluded because gestation does not alter the gynecological management of exams if the patient has any precursor lesions. In addition, the specialist questioned the use of the age of screening in the algorithms and suggested that these included only the type of lesion and that two algorithms were removed from the guideline because they were equivalent. A3 suggested that the ASC-US algorithm were revised, but did not specify which aspect needed revision. Regarding the age for onset of screening, duly justified throughout the text of the clinical guideline, the authors did not carry out the suggested change because it is only a textual presentation form, which actually facilitates the identification of the target audience of the guideline.

The expert A3 assigned 5 points to the item 16, justifying that the algorithm exposed on page 20 was ambiguous. The algorithm was revised. In the item 17, A4 attributed 3 points to this item, justifying that the key recommendations needed to be more concise and objective. Considering that the topics covered were already concise in relation to the topic addressed, we chose not to make any further cuts or shortenings in the text of the clinical guideline.

Regarding the *domain 5*, A1 attributed 5 points to the item 18, justifying that there is little availability in the Brazilian public service for the use of more expensive methods to complement the screening of cervical cancer in the population. The specialist A3 attributed 4 points to the items 20 and 21, but made no comments on this decision. A2, A3 and A4 assigned 1, 4 and 3 points, respectively, to the item 21. A2 said criteria for monitoring/auditing the guideline were not present; A3 did not comment on this item, and A4 suggested a better approach to the audit of the clinical guideline. After the review of these suggestions by the authors, it was agreed to create monitoring criteria and the need to carry out a future study, after using the clinical guideline for a certain period of time, in order to evaluate its application.

Regarding the *domain 6*, the specialist AE4 attributed 1 point to the item 23, justifying not having identified the conflicts of interest among the team members that prepared the clinical guideline. The authors took this suggestion into account and wrote more explicitly the lack of conflict of interest among the members that developed the clinical guideline. It is noteworthy that this study was funded by the National Scientific Council of Technological Development (CNPq) under process nº 479373/2013-2.

As for the overall assessment of the guideline by the four experts who participated in the study, the scores varied between 5 and 6 points. As for the question: “Would I recommend the use of this guideline?” present at the end of the AGREE II, all the experts answered “yes, with modifications”. The recommendations of the specialists in relation to the clinical aspects and those intrinsically related to the implementation of the clinical guideline in the reality of the study site were accepted.

## Discussion

This study brings unprecedented results regarding the elaboration of clinical protocols in the nursing area, especially in the topic of women’s health. In the search carried out in PubMed, only 212 articles that used the instrument AGREE II in the health area were available. From these, only 186 corresponded to publications in the last 5 years, only 112 used the AGREE II to evaluate clinical protocols, and 74 consisted in systematic reviews of protocols. Only one study referred to Nursing^15^ and none addressed the development of clinical protocols in gynecology.

The protocol presented herein proposes the implementation of new technologies in a standardized clinical decision-making process to prevent cervical-uterine cancer to be adopted in primary health care, contributing to a more efficient conduct of the professionals who use it. This aims to directly affect the incidence and morbimortality of the disease.

The inclusion of physicians and nurses in the evaluation of this clinical guideline was important given the diversity of opinions and clinical approach inherent in each professional category. Both professionals work in the same area and converge to reach the same goal, which is the reduction of morbidity and mortality from cervix-uterine cancer. Thus, the developed clinical guideline has applicability in gynecological health care among multiprofessional teams, considering that it was validated by different professional categories and, therefore, contemplates its main purpose: to be a practical guide to screening actions for cervical cancer to be used by professionals working in this area, in the scope of primary health care.

The evaluation of clinical guidelines by an interdisciplinary team is supported by the AGREE II, which has been used in other studies in order to achieve a positive and comprehensive evaluation^16^ ^-^ ^17^. Furthermore, a clinical guideline built by an interdisciplinary team to be used in a specific area of ​​the health service becomes more objective, capable of directing the professionals towards effective clinical decision-making, and helps avoiding multiple clinical judgments about health problems^18^ ^-^ ^19^.

Although one of the evaluators (A3) had suggested removing the digital cervicography from the protocol, the authors did not accept this suggestion, because there are studies that prove the efficacy of this method during clinical consultations in gynecology and that because this method serves the purpose of tracking cervical cancer precursor lesions. A study conducted in Korea in private clinics with 1547 patients showed a positive correlation between the diagnoses revealed by digital cervicography and by cytopathological examination, in which both identified equivalent cervical cancer precursor lesions^20^.

A study that aimed to build a clinical protocol for diabetes mellitus and also used AGREE II presented lower indices than those found in our study (Domain 1-66.7%, Domain 2-35%, Domain 3-36.5%, Domain 4-61.5%, Domain 5-27% and Domain 6-40%)^21^. Although the AGREE II does not establish a cut-off point for guideline quality, it is worth noting that this clinical guideline was evaluated by 16 judges and recommended by 12 of them^22^. AGREE II recommends the evaluation by 4 experts only, and the strategy used to calculate adequacy was designed for 4 evaluators. It is known that the greater the number of appraisers, the greater is the diversity of opinions and the greater the possibility of generating disagreement between them, which may explain the low adequacy indices found in the above mentioned diabetes protocol.

A suggestion of classifying the quality of clinical guidelines was adopted by the authors of a study carried out in Spain, which established the following classification for quality of clinical protocols: percentage of suitability less than or equal to 25% was considered very low; suitability equal to 50% was low; suitability between 50% and 75% was high; and suitability above 75% was very high ^22^. This is in line with the present study, for it was established here that a clinical protocol should obtain a minimum of 75% adequacy in its domains to be considered of good quality.

## Conclusion

The clinical guideline studied brings technological innovations regarding the screening of lesions that cause cervical cancer, such as digital cervicography and colposcopy. The study was evaluated according to the AGREE II and obtained scores consistent with a good quality guideline, which can be implemented in health services in order to improve gynecological health care. Among the limitations of the study is the fact that the study was related to actions that occurred in a single research locus, which reduces its geographical coverage in relation to the target population and its power of inference to other primary health institutions. The realization of a clinical study is recommended to analyze the impact and the implementation of cervical cancer screening tests within a set period of time to verify the cost-effectiveness of the use of this clinical guideline in order to investigate the viability of its implementation in the routine of health services, so that the guideline may be widely adopted in health units. The guideline will be updated periodically in order to preserve actions based on high levels of evidence and better recommendations.
